# Increasing land-use intensity reverses the relative occupancy of two quadrupedal scavengers

**DOI:** 10.1371/journal.pone.0177143

**Published:** 2017-05-11

**Authors:** Joshua P. Twining, Henry Bernard, Robert M. Ewers

**Affiliations:** 1 Department of Life Sciences, Imperial College London, London, United Kingdom; 2 Institute for Tropical Biology and Conservation, Universiti Malaysia Sabah, Kota Kinabalu, Malaysia; U.S. Geological Survey, UNITED STATES

## Abstract

Human land use is continuously altering the natural environment, yet the greater ecological implications of this change for many groups that are key to healthy ecosystem functioning remains uncharacterised in the tropics. Terrestrial scavenging vertebrates are one such group, providing integral ecosystem services through the removal of carrion which is a crucial component of both nutrient cycling and disease dynamics. To explore how anthropogenic processes may affect forest scavengers, we investigated the changes in the relative occupancy of two important terrestrial scavengers along a gradient of land use intensity, ranging from protected forest to oil palm plantation in Borneo. We found the Malay civet (*Viverra tangalunga*) had highest, albeit variable, occupancy in areas of low land use intensity and the Southeast Asian water monitor *(Varanus salvator macromaculatus)* had highest occupancy in areas of high land use intensity. Land use had no effect on the combined occupancy of the two species. In high land use intensity sites, individual water monitors were larger and had better body condition, but at population level had a highly biased sex ratio with more males than females and increased signs of intraspecific conflict. We did not assess scavenging rate or efficiency as a process, but the high occupancy rates and apparent health of the scavengers in high land use intensity landscapes suggests this ecological process is robust to land use change.

## Introduction

Scavengers play vital roles in nutrient cycling and redistribution[1] as well as disease dynamics via the removal of carrion from the environment, providing a crucial ecosystem service[2]. The disruption of intact scavenger communities has possible far reaching implications and understanding how scavenging communities function and respond to environmental changes has become a subject of increased importance [3]. Scavenging as a process has often been overlooked [1]-[4], possibly due to human aversion to rotting substances in combination with the ephemeral nature of carrion, which has led to a paucity of information on scavenger communities, ecology and dynamics[5]-[6],. A high percentage– 60 to 100%–of naturally occurring carrion is utilised and removed by vertebrate scavengers rather than microbes and arthropods[7]-[8]-[9]-[10],. Recent studies targeting vertebrate scavengers have been predominantly conducted in relatively pristine environments in temperate or savanna regions [1]-[2]-[4]-[6]-[11]-[12], with few examples from tropical forests [6]. Consequently, we have little information about how habitat and human disturbance influence scavenger communities [13], and that knowledge gap is most pronounced for tropical ecosystems that are undergoing dramatic levels of anthropogenic land use change.

The Southeast Asian island of Borneo has very high rates of deforestation and habitat loss, currently estimated to have just over 50% of natural forest cover remaining[14]-[15]. This is down from more than 75% cover in 1973. The highest losses over this period were recorded in the Malaysian state of Sabah which lost almost 40% of its total forest [16]. The primary driver of this relentless deforestation in Sabah, is at present, the proliferation of the oil palm industry. By 2007 the oil palm industry accounted for 5% of Malaysia’s gross national income and that proportion has continued to grow rapidly since [17]. Such economic incentive puts tremendous pressure on the remaining shrinking forests of Borneo. Despite oil palm accounting for 60% of vegetable oil production globally in 2008, it was the topic of only 10% of total vegetable oil research [18]-[19], meaning relatively little is known about the ability of species to persist in the degraded and fragmented habitats left behind following the expansion of oil palm plantations, and the changes such degradation will have on community composition, interspecific interactions and ecosystem functioning. This is particularly true of vertebrate scavenger species, whose population responses to land use change remain largely uncharacterised.

The extirpation of scavenger species from localised ecosystems can disrupt the composition and, therefore, the efficiency of scavenging communities [20]-[21]-[22]. The inability of a key scavenger species to persist in a degraded environment can not only alter and interrupt the flow of energy in an ecosystem, but may also lead to increased health risks for people and other wildlife through the persistence and spread of disease due to prolonged presence of carrion [6]. Previous research in temperate zones suggests that with land use driven habitat transformation, mesopredator species may increase in abundance and compensate for the loss of apex scavengers and maintain the rate of carrion removal, however results were equivocal[12] and do not necessarily translate to tropical ecosystems.

In the present study we provide currently lacking information on the effects of tropical land use change on two of the dominant terrestrial scavenger species in Borneo. In northern Borneo, the terrestrial scavenger species include Southeast Asian water monitors *(Varanus salvator macromaculatus)*, Malay civets *(Viverra tangalunga)*, Sun bears *(Helarctus malayanus)*, Oriental small-clawed otters *(Aonyx cinera)*, Smooth-coated otters *(Lutrogale perspicillata)*, Bornean bearded pig (*Sus barbatus*) and Short-tailed mongooses *(Herpestes brachyurus)* [23]-[24]. Of these species, Malay civets and Southeast Asian water monitors are the two most common scavenger species at our study site [25]Malay civets and Southeast Asian water monitors share a similar ecological niche; both are generalists, and they typically live sympatrically, albeit with a temporal separation of activity in that civets are nocturnal while monitors are diurnal[26]-[27]-[28]. Southeast Asian water monitors (*Varanus salvator)* on Tinjil Island, Indonesia showed a significant positive correlation between human disturbance and relative abundance [29], and so we expect the abundance of *V*. *s*. *macromaculatus* to be highest in areas of highest land use intensity. Malay civets (*Viverra tangalunga)* in Riau Province, Sumatra, Indonesia showed a relatively low occupancy and detection rate in oil palm, decreasing as distance from forest edge increases [30], whereas another study in Sabah, Borneo, showed no significant difference between occupancy of Malay civets in forest or oil palm [31]. This latter result, however, may be due to the close proximity (<1 km) of the oil palm sampling point to the forest edge, and we therefore expect abundances of *V*. *tangalunga* to be lowest in areas of increased land use intensity.

A species’ ability to perform ecological functions is not just related to their abundance; many other features of a population, such as age and size structure, exert strong determinants on a species’ functional importance [12]. Research evaluating the complex effects, and the far reaching ecological implications, of altered structure and phenotypes of wild populations is lacking [32]-[33]. We therefore go beyond examining the abundance of scavenger species and conduct a more detailed examination of the population structure, phenotypic parameters and health of the animals that form the population. We focus this investigation on the Southeast Asian water monitor, primarily because it is possible to capture and process individuals without anaesthesia and the possible complications and long term health effects which come with administering anaesthetics to wild animals [34]. The Southeast Asian water monitor is one of the largest, and most widely distributed, squamate lizard complexes in the world [35]-[36]-[37]. In Sabah they are mesopredators, inhabiting forested and open habitats alongside numerous carnivorous mammalian species [23]. Little is known about the functional role of monitor lizards in many ecosystems, as their impact has previously been omitted in favour of studying placental mammals [38]. We expect to find sites with high land use intensity to have populations characterised by larger body masses and greater aggregations of males, as observed in Lace monitors [39]. However, not all effects of land use intensity are so straightforwardly predictable: environmental factors may also have negative effects such as increasing the abundance of parasites, perturbations to naturally occurring sex ratios and increasing territorial conflict which would have obvious detrimental effects on the health of individuals and communities as a whole.

## Methods

### Study site

This project was conducted at the Stability of Altered Forest Ecosystems (SAFE) Project: an experimental landscape situated in the North-East Malaysian state of Sabah [40]. The SAFE project is based in a 7,900 hectare landscape that is currently being converted from logged forest into oil palm plantation, experimentally setting the size and location of remnant forest fragments and riparian strips to monitor the effect of forest fragmentation and riverine margin size on a wide range of ecosystem processes [40].

We sampled the two species of vertebrate scavengers along streams in six watersheds that encompassed a gradient of land use intensity. Our lowest land use intensity site was inside a 2,200 ha Virgin Jungle Reserve (VJR) located immediately adjacent to the SAFE project. The VJR is characterised as primary forest by local government, although it has had some illegal logging on the lower slopes. At the other extreme of land use intensity, we sampled inside an existing oil palm plantation (OP) that was planted in 2006[40]. At intermediate levels of land use intensity, we sampled in four watersheds within forest that has undergone two rounds of selective logging, with the last round occurring between 2000 and 2008. All watersheds were approximately equal in terms of area (mean = 260 ha, SD = 10 ha) and slope (mean = 16°, SD = 2°), and contain headwater streams of approximately 2 km in length [40].

To quantify land use intensity, we utilised RapidEyeTM satellite images acquired over the SAFE landscape in 2012 and 2013. Images were combined with data from 193 vegetation plots to generate upscaling algorithms mapping aboveground biomass (AGB) at 30 m resolution over the entire study area [41]. We extracted data from the upscaled maps that fell within a 100 m buffer either side of stream beginning at the most downstream location we sampled and continuing to the head of the stream. For each stream, we calculated the average AGB within the buffer zone, which we used an index of land use intensity. Low intensity sites such as in the VJR have high values of AGB, whereas high intensity sites such as oil palm plantation have very low values of AGB.

### Sampling method

Sampling was conducted during two site visits: January 2014 to June 2014 and again in December 2014 to March 2015. Additional salvage logging operations were operating in the area during the second site visit as the landscape is actively being converted from logged forest to oil palm plantation [40], and we did not collect new data from two of the watersheds where land use intensity changed between the first and second visit.

The two focal vertebrate scavengers were censused using large baited cage traps (150 x 50 x 50 cm). Traps used were designed and custom made for the investigation in which the door was held open by a hook that was connected to the bait, using such a mechanism that only trapped individuals when bait had been removed to ensure actual incidents of carrion removal were being recorded, rather than simply records of animals entering traps or investigating scent [42]. It should be noted that such a trapping methodology does not represent the full community of scavengers–it will not record trap-shy species and is inappropriate for quantifying carrion removal by birds, for example–but it is an appropriate method to sample the two target species we were specifically interested in. To understand the degree of bias between animals we trapped and the wider, potential scavenging community, we deployed camera traps in an *ad hoc* manner concurrently with cage traps. On any given sampling day, up to five camera traps were set, each facing a separate cage trap and programmed to be triggered by movement, at which point they captured six images over a one minute period.

Each site had five traps at approximately 250 m intervals along the stream. Traps were baited with carrion between 07:00 and 09:00 in the morning to target scavenger species specifically, and checked daily at the same time period for between seven and 10 consecutive days before we moved onto the next site. Bait was randomised with a single bait used per trap, and included whole native fish or dead rodents, halved domestic chickens, and pig. Capture rates were not significantly influenced by bait type (binomial general linear model (GLM), *p* = 0.313), and neither was there a significant bait × species identity interaction (binomial GLM, *p* > 0.999). Bait type was, therefore, not included as a predictor in further analyses. All bait items were > 1 kg to remove the effect of ontogenic shifts in feeding preferences observed in Southeast Asian water monitors[43]-[44]. Bait was deployed fresh and replaced every 48 hours if not consumed by a scavenger to remove adverse effects of decomposition. Watersheds were sampled on between one and five sampling periods giving an average sampling effort of 133 trap nights per watershed, distributed over the two site visits (range = 50–190).

Captured mammals were identified to species and released. Water monitors captured for the first time had their head covered by a black cloth to induce an unconscious state that reduces stress and facilitates processing [42]. We injected each with a unique PIT tag inserted in the thigh of the left hind leg, inserting it between muscle and skin pointing downwards [45] for future identification. Tagged individuals were given a tail crest clipping in order to facilitate rapid identification of re-captures during subsequent trapping days. Captured individuals had three morphometric measurements recorded: snout-venter length, body mass, and head length. Sex of individuals was discerned by observing the presence of hemipenal eversion under duress [46]; although this is the simplest morphological approach it was the most suitable as cloacal probing is prone to inaccurate results due to presence of hemiclittoral sacculae at approximately the same position of male hemipenes [47], and hemipenal transillumination methods are only applicable in smaller varanid species [48]. Body condition indices were calculated by dividing loge-transformed mass by loge-transformed snout-vent length, following the methodology from Jessop *et al*. (2012) [39]. Transforming data reduces the influences of ontogenic body shape changes and eliminates allometric scaling effects. Individuals were searched for ectoparasites residing on the scales and endoparasites in the mouth and cloacal opening [42]. Finally, individuals were checked for scarring from previous intraspecific competition. Scars of varying size and age were considered of equal ‘importance’ and counted as such. Previously captured Southeast Asian water monitors were identified from the embedded PIT tag and immediately released. Any injuries resulting from trap doors and tag injectors were disinfected thoroughly with iodine before releasing animals.

### Data analysis

We attempted to generate capture-mark-recapture (CMR) density estimates for the Water monitor, but did not have sufficient sample size for all sites and were therefore unable to obtain rigorous estimates across the land use gradient. Moreover, we were unable to use CMR for the Malay civets for which we did not have data on individuals. Consequently, we discarded the use of CMR in favour of relative occupancy. Occupancy of single species were modelled using generalised mixed effects model (GLMMs) with binomial errors and two random effects: trap identity nested within river and day nested within sample period. Analyses were performed using R Version 3.2.1 with the lme4 package [49]-[50]. For all response variables, we used aboveground biomass as the sole predictor variable and assessed parameter significance using likelihood ratio tests, comparing the fitted model to an intercept-only null model that retained the full set of random effects. We chose not to test for the statistical significance of random effects as they form a component of our experimental design rather than a focus of our study [51]. We also chose not to adjust our occupancy estimates for detectability. This is because our aim was to investigate the effects of environmental factors on incidence rates rather than make an absolute estimate of abundance or species richness, in which case it is unnecessary to control for detection probabilities [52]. We modelled relative occupancy for each of the two target species separately, as well as for the combined occupancy of the two species.

Water monitor phenotypic and community health parameters were analysed using linear regression (body mass, snout-venter length, head length and body condition), with data log^10^-transformed to improve model residuals where appropriate, or generalised linear models with either binomial errors (sex and presence of scars) or poisson errors (number of ectoparasites and number of endoparasites). As with the occupancy analysis, aboveground biomass was used as the sole predictor variable.

### Ethics approval

Sabah Biodiversity Centre provided all necessary permits for field work and data collected.

SAFE Science Advisory Committee provided feedback on study design.

We followed all guidelines outlined by the Home Office, Animals (Scientific Procedure) Act 1986 (ASPA). It states "the ringing, tagging or marking of an animal, or the application of any other humane procedure for the sole purpose of enabling an animal to be identified, is not a regulated procedure if it causes only momentary pain or distress and no lasting harm." ASPA relates to regulated procedures and ethics, “A procedure is regulated if it is carried out on a protected animal for a scientific or educational purpose and may cause that animal a level of pain, suffering, distress or lasting harm equivalent to, or higher than, that caused by inserting a hypodermic needle according to good veterinary practice” (Home Office, 2016).

## Results

### Occupancy rates of terrestrial scavengers

We had 118 captures of individuals belonging to four species in a total of 791 trap nights, with the most abundant scavengers captured being the Malay civet (*N* = 61 captures) and the Southeast Asian water monitor (*N* = 51). We also captured Domestic dogs *Canis familiaris* (*N* = 5) and the Collared mongoose *Herpestes semitorquatus* (*N* = 1). There were 28 incidences of carrion removal where we failed to capture the animal responsible; capture failures were not correlated with our measure of land use intensity, aboveground biomass (binomial GLMM, χ^2^ = 0.61, *P* = 0.433). Observations from camera traps indicated carrion had been disturbed by a further five species that we did not capture: Asian elephant *Elaphus maximus*, Bornean bearded pig *Sus barbatus*, Malayan sun bear *Helarctos malayanus*, Smooth-coated otter *Lutrogale perspicillata*, Short-tailed mongoose *Herpestes brachyurus*, and Gold-ringed cat snake *Boiga dendrophila*.

**Fig 1:**
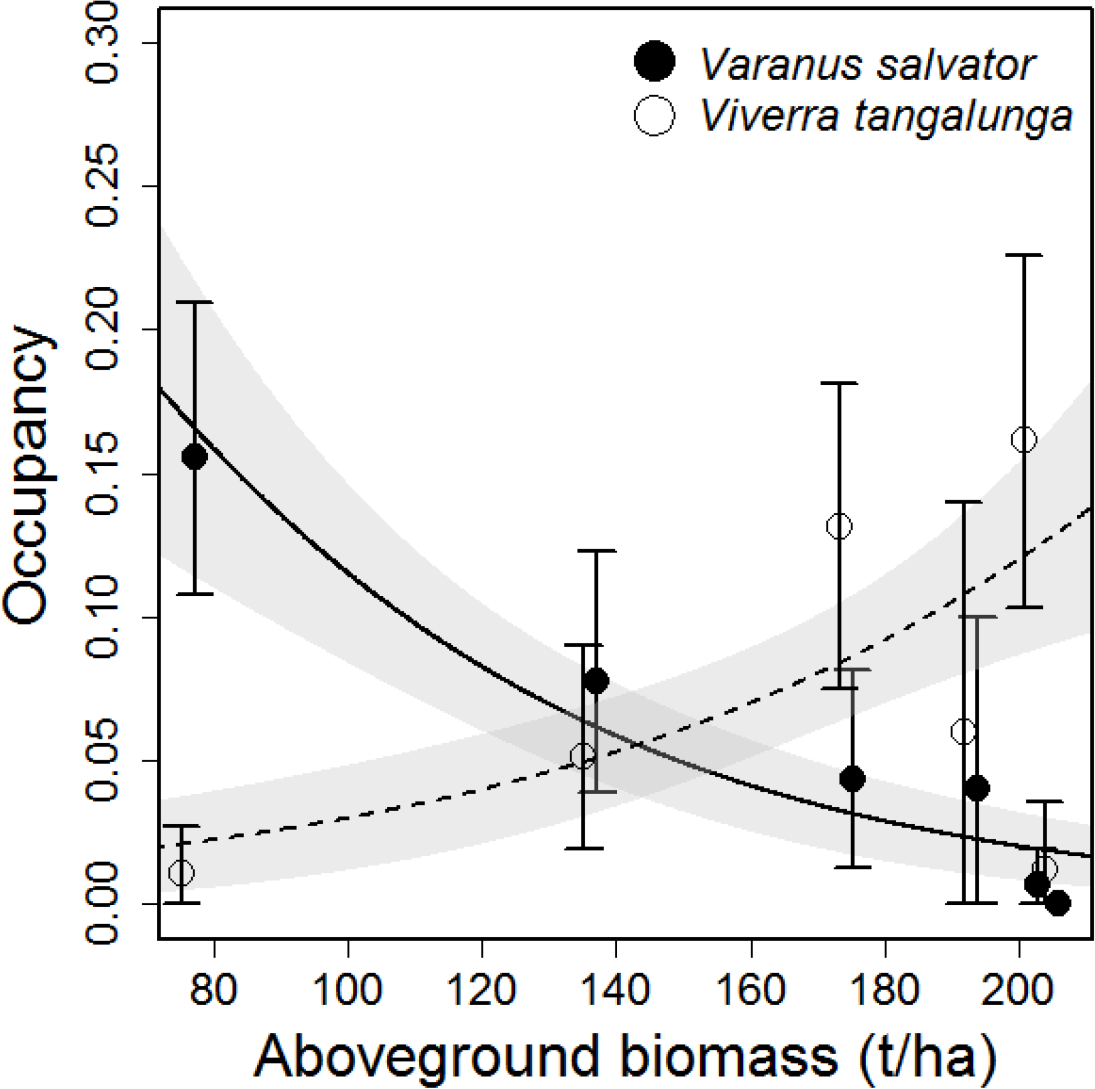
Occupancy patterns of the Malay civet (*Viverra tangalunga*) and the Southeast Asian water monitor (*Varanus salvator macromaculatus*) along a gradient of land use intensity. Land use intensity is represented by aboveground tree biomass, with low values representing intensive land use in oil palm plantation, intermediate values representing logged forest of different intensities, and high values representing primary forest. Error bars represent the 0.025 and 0.975^th^ quantiles estimated from 1000 bootstrapped samples of the data. Fitted lines are from a binomial generalised linear model fitted through the raw data. Dashed lines indicate non-significant patterns and shaded polygons represent the 95% confidence interval.

Aboveground biomass explained significant portions of the variance in occupancy of Southeast Asian water monitors (Fig 1, binomial GLMM, χ^2^ = 16.82, *P* < 0.001), but not for Malay civets (Fig 1, binomial GLMM, χ^2^ = 2.35, *P* = 0.125). Occupancy of Malay civets exhibited a tendency to be lowest in oil palm plantation but was highly variable in forest with high aboveground biomass (Fig 1), with some sites having very high occupancy and others having very low occupancy. Southeast Asian water monitors demonstrated a consistent trend, having highest occupancy in the most disturbed land use areas and decreasing occupancy with increasing tree biomass (Fig 1). Combined occupancy of the two species together exhibited no pattern along the gradient of aboveground biomass (binomial GLMM, χ^2^ = 1.14, *P* = 0.286).

### Water monitor phenotypic parameters and community health

**Fig 2:**
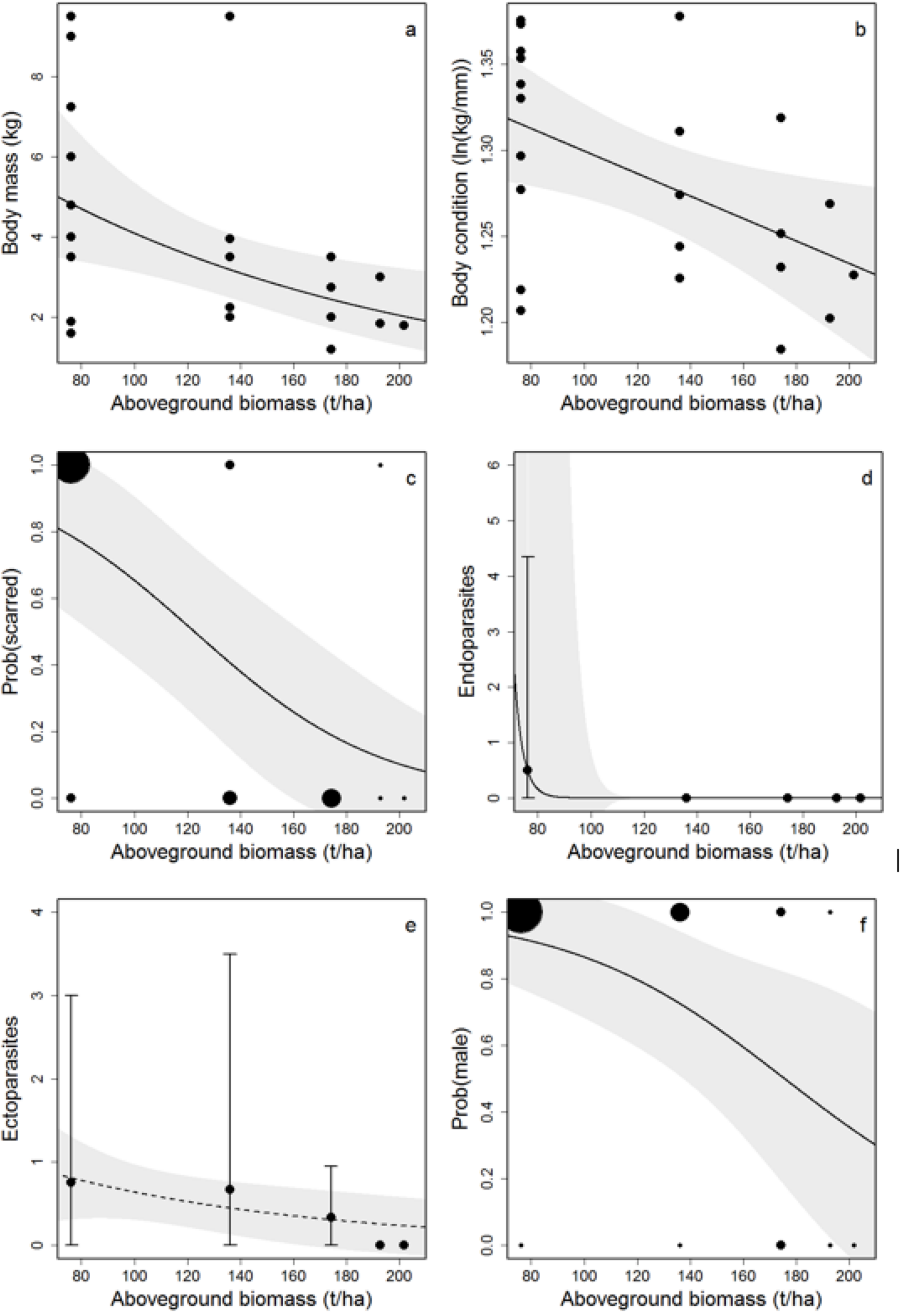
Effect of land use intensity on the phenotype, health and population structure of water monitors (*V*. *s*. *macromaculatus*). Land use intensity is represented by aboveground tree biomass, with low values representing intensive land use in oil palm plantation, intermediate values representing logged forest of different intensities, and high values representing primary forest. Southeast Asian water monitors in high intensity land uses tend to have larger body size (a) and better body condition (b), but have a higher probability of being scarred (c) and have more buccal nematodes (d). They do not have higher ectoparasite loads but are more likely to be male than female (f). Point size in panels (c) and (f) are linearly scaled to represent the number of individuals with similar values. Fitted lines are from linear models (a,b) binomial generalised linear models (c,f) or poisson generalised lineary models (d,e) fitted through the raw data. Dashed lines indicate non-significant patterns and shaded polygons represent the 95% confidence interval.

Southeast Asian water monitors tended to be larger in more disturbed locations, with significantly negative relationships between aboveground biomass and log_10_-transformed body mass (linear regression, *F*_1,20_ = 8.09, *P* = 0.010) (Fig 2), snout-vent length (linear regression, *F*_1,20_ = 8.34, *P* = 0.009) and head length (linear regression, *F*_1,18_ = 10.16, *P* = 0.005). Individuals in disturbed locations also tended to have better body condition than those in less disturbed locations (linear regression, *F*_1,20_ = 6.93, *P* = 0.016), despite having a higher frequency of scarring (generalized linear regression, *P* = 0.007) and higher numbers of endoparasites (generalized linear regression, *P* = 0.004) (Fig 2). All identified endoparasites were buccal nematodes belonging to *Pentastomids* spp, and all were collected from a single individual in the oil palm plantation that had six endoparasites while none were found in any other individuals. A total of 14 ectoparasites were detected on five individuals, and all ectoparasites were the tick *Amblyomma helvolum*, and there was no statistically significant effect of aboveground biomass on the number of ectoparasites (generalized linear regression, *P* = 0.124). The population sex ratio also varied along the land use gradient, with populations in less disturbed locations having sex ratios of approximately 1:1 males:females but the ratio exceeding 4:1 in the most heavily disturbed locations (generalized linear model, *P* = 0.028) (Fig 2).

## Discussion

Anthropogenic land uses are increasingly altering natural habitats, but we found that the combined occupancy of two dominant terrestrial scavengers is robust to even dramatic changes in land use such as the conversion of forest to oil palm plantation. We did, however, find evidence that disturbance of riverine margins results in a significant shift in the relative occupancy of the two dominant scavenger species to the ecosystem function, with the Southeast Asian water monitor becoming more abundant as disturbance increased. While we did not find a statistically significant reduction in the occupancy of the competing Malay civet, it was nonetheless very rarely encountered in the most intensive land use, suggesting the Southeast Asian water monitor is filling a niche created by localised reduction in abundance of otherwise dominant mammalian scavengers. These results are consistent with other studies documenting significant shifts in scavenger community composition in fragmented landscapes in temperate zones, where modified landscapes provide insufficient habitat for the persistence of top trophic level predators while providing increased food resources and decreased interspecific competition for generalist species [12]-[53]-[54].

Our finding that the total occupancy of the two dominant terrestrial scavengers is unaffected by land use intensity is an important one, suggesting as it does that land use intensity may not result in a change to the rate at which carrion is removed. This does, however, make an explicit assumption that occupancy of the two species correlates closely with their scavenging rates, which is not something we have directly quantified. Moreover, it assumes that any given individual consumes carrion at the same rate, which is unlikely given our findings of significant shifts in the body mass of Water monitors along the land use gradient.

We demonstrated that increasing land use intensity results in significant changes to the morphological and phenotypic parameters of the Southeast Asian water monitor population. Individuals from high land use intensity areas had longer lengths, larger masses and better body condition than in low land use intensity sites. This is expected as Water monitor populations in areas of high anthropogenic pressures experience reduced inter-specific competition while simultaneously accessing a new resource in the form of human trophic subsidies (human refuse, domesticated animals, and edible products of agriculture)[29], [55]. Initially this suggests that these individuals are in better health than those in less disturbed locations but that is, however, at least partially offset when considering the increased level of scarring, the skewed sex ratio and the weak evidence for increased parasite loads. These adverse effects are more difficult to empirically quantify than body size distributions, but are just as valid in assessing the health of a population [39]. Individuals from areas of high land use intensity were in better morphological health, but highly biased sex ratios increase sexual competition for mates and can potentially decrease overall reproductive success[56]. Increased scarring is indicative of elevated social conflict and territoriality resulting from high density[42]-[57] and increased parasite load can directly reduce overall fitness [39]-[58]. In addition, the cannibalistic nature of varanids [46], in combination with higher aggregations of individuals, may result in high levels of juvenile mortality. If high intensity land-use habitats attract individuals from natural forests and promote survival through increased phenotypic quality, then offspring will likely be subjected to elevated levels of predation as well as being faced with the adverse effects of highly skewed sex ratios. In the long term this may drive population decline, resulting in an ecological trap[59]. To quantify this, however, would require a long term investigation into the population dynamics of these lizards and knowledge of immigration and mortality rates. Without these, a decisive measure of fitness is unobtainable and the consequences of anthropogenic land-use on long term varanid population health remains equivocal.

Our data were collected in riverine margins where land use intensity last changed when the logged forest underwent a second round of salvage logging between 2000 and 2008[60]. This situation was, however, changing during our second period of sampling, with additional salvage logging operations present in the area as the landscape is converted from logged forest to oil palm plantation [40]. It remains to be seen whether this new disturbance will lead to further short or long term changes to the relative occupancy of these two species. Our informal, qualitative observations suggest this probably will occur, as large and medium sized mammals appear to be moving away from the disturbance but Southeast Asian water monitor populations have not yet benefited from it.

Our data have shown that land use intensity alters the relative occupancy of two key terrestrial scavenger species, which we hypothesise is caused by localised extirpation of the mammalian scavengers and top predators that inhabit low land use intensity landscapes. The individual scavengers in high land use intensity areas appear to be in good phenotypic health with large body sizes and good body condition, despite the population having a heavily biased sex ratio, increased sexual and territorial competition and potentially higher parasite loads. The apparent health of Water monitor populations in high land use intensity areas likely contribute to the maintenance of carrion removal rates in these agricultural landscapes.
